# Supplementary material for: The Multifaceted Syndromic Primary Immunodeficiencies in Children
Source: J Clin Med. 2023 Jul 28;12(15):4964. doi: 10.3390/jcm12154964 (PMC10419544; doi:10.3390/jcm12154964)
Supplement: Supplementary file 1 [file jcm-12-04964-s001.zip › Table S2.pdf]

Patient number	Diagnosis	Immunosuppressive, antiinflammatory and biologic modifier therapy	Indications	Response
5	DiGeorge syndrome	rituximab, prednisolone, mycophenolate mofetil	Autoimmune thrombocytopenia, lymphopenia and neutropenia	partial
6	DiGeorge syndrome	bortezomib, rituximab, prednisolone, sirolimus, mycophenolate mofetil	Autoimmune haemolytic anaemia	partial
8	DiGeorge syndrome	rituximab	EBV lymphoroliferative disease	good
9	DiGeorge syndrome	prednisolone, cyclosporin, mycophenolate mofetil, tacrolimus	Autoimmune haemolytic anaemia, thrombocytopaenia and neutropaenia	poor
		prednisolone, cyclosporin, tacrolimus	Autoimmune hepatitis	poor
		prednisolone, rituximab, cyclophosphamide	EBV negative lambda light chain restricted polymorphic B cell lymphoproliferative disorder	good
10	Trichohepatoenteric syndrome	prednisolone, baricitinib, mycophenolate mofetil	Chronic diarrhoea, IBD-like histology	partial
11	Trichohepatoenteric syndrome	mycophenolate mofetil	Chronic diarrhoea	partial
17	Sideroblastic anaemia with B cell immunodeficiency, periodic fevers and developmental delay (SIFD syndrome)	anakinra, colchicine	Autoinflammation	poor
	Sideroblastic anaemia with B cell immunodeficiency, periodic fevers and developmental delay (SIFD syndrome)	etanercept, colchicine	Autoinflammation	good
21	Sideroblastic anaemia with B cell immunodeficiency, periodic fevers and developmental delay (SIFD syndrome)	anakinra	Autoinflammation	good
30	Sideroblastic anaemia with B cell immunodeficiency, periodic fevers and developmental delay (SIFD syndrome)	etanercept	Autoinflammation	good
31	Wiskott-Aldrich syndrome	prednisolone, rituximab	Autoimmune thrombocytopaenia	poor
