# Supplementary material for: The Multifaceted Syndromic Primary Immunodeficiencies in Children
Source: J Clin Med. 2023 Jul 28;12(15):4964. doi: 10.3390/jcm12154964 (PMC10419544; doi:10.3390/jcm12154964)
Supplement: Supplementary file 1 [file Table S1.pdf › Table S1.pdf]

Patient	Genetic disorder	Age (years)	Sex	Immunophenotypic abnormalities	Infections	Non-infectious clinical features	Immunoglobulin replacement	Antibiotic prophylaxis	Immunosuppresive therapy	Other treatment	Mortality	Cause of death
1	Ataxia telangiectasia	16.3	Female	Low T and B cells, low IgG, A and M.	Recurrent respiratory infections, ear infection, toe nail fungal infection.	Ataxia, speech problem, nystagmus, eye teleangiectasia, skin pruritus, thoracolumbar scoliosis, fatty liver, gallstones, gallbladder polyps, transaminitis, obesity, vitamin D deficiency, impaired glucose tolerance.	Yes	Yes			No	
2	Ataxia telangiectasia	12.6	Female	Low T and B cells, low IgG and A.	Recurrent respiratory infections, impetigo.	Transaminitis, nystagmus, fatty liver, slow learner, delayed motor skills, bulbar conjunctival telangiectasia, scoliosis.	Yes	Yes			No	
3	Ataxia telangiectasia	20.3	Male	Low T cells, low IgG, A and M.	Pneumonia, neutropaenic sepsis	Right cerebral infarction, cerebral venous sinus thrombosis, calf muscle tightness, vitamin D deficiency, transaminitis, lymphoma.	Yes	No	Chemotherapy		No	
4	Ataxia telangiectasia	22.9	Male	Low T and B cells, low IgG, A and M.	Pneumonia, sepsis.	Avascular necrosis of both hips, vitamin D deficiency, long QT syndrome, dystonia, thoracolumbar scoliosis, skin striae, myopia, lymphoma.	Yes	No	Chemotherapy		No	
5	DiGeorge syndrome	12.8	Male	Low T, B and NK cells, raised double negative T cells, low IgG and G4, inadequate vaccine repsonses, autoimmune neutropenia, lymphopaenia and thrombocytopenia, MBL deficiency, allergic reaction to vancomycin and penicillin.	Bronchiectasis, recurrent respiratory infections, recurrent skin infections, subacute bacterial endocarditis, gastroenteritis, *C. difficile* infection.	Truncus arteriosus, ventricular septal defect, developmental delay, learning difficulty.	Yes	Yes	Rituximab, prednisolone, mycophenolate mofetil		Yes	NA
6	DiGeorge syndrome	13.3	Male	Severe combined immune deficiency, absent T cells, absent PHA response, low IgG, A and M, autoimmune haemolytic anaemia, alopecia areata, autoimmune thrombocytopaenia.	Oral candidiasis, recurrent respiratory infection, *C. difficile* infection, sapovirus, astrovirus and norovirus gastrointestinal infection, enterovirus infection, panniculitis.	Hypoparathyroidism, femoral fracture, scoliosis, genu valgum, raised alanine transaminase, hepatomegaly, iron overload due to frequent transfusion, myopic astigmatism, alopecia areata, developmental delay, tetralogy of Fallot, pulmonary artery stenosis, urinary calculus, developmental delay.	Yes	Yes	Bortezomib, Rituximab, prednisolone, sirolimus, mycophenolate mofetil	Thymic transplant	No	
7	DiGeorge syndrome	21.9	Female	Low B cells, IgG2 and G4, IgA, IgM. Autoimmune hypothyroidism. Impaired vaccine response.	Bronchiectasis.	Cleft palate, pulmonary stenosis, ventricular septal defect, learning difficulty, ADHD, primary nocturnal enuresis, vitiligo, thoracic scoliosis, autoimmune hypothyroidism, developmental delay.	Yes	Yes			No	
8	DiGeorge syndrome	21.4	Male	Low B cells, IgG, A and M, poor vaccine repsonses, autoimmune cytopaenias (neutrophil, platelet, anaemia), EBV driven T and B lymphoproliferative disease.	Bronchiectasis, otitis externa.	Cleft palate, iron deficiency anaemia, speech delay, learning difficulties, developmental delay.	Yes	Yes	Rituximab		No	
9	DiGeorge syndrome	23.0	Female	Autoimmune haemolytic anaemia and thrombocytopaenia, autoimmune hepatitis, polymorphic B cell lymphoproliferative disease, low B cells, IgG and M (following lymphoproliferative disease treatment).	Groin infection, otitis media, cellulitis.	Interrupted aortic arch, ventricular septal defect, tricuspid regurgitation, bicuspid valve, cleft palate, lymphangitis, liver fibrosis, learning difficulties, allergic rhinitis.	Yes	Yes	Prednisolone, mycophenolate mofetil, cyclosporin, tacrolimus, Rituximab, cyclophosphamide.		No	
10	Trichohepatoenteric syndrome	5.1	Male	Poor vaccine responses, interferonopathy.	Recurrent respiratory infections.	Caecal perforation, inflammatory bowel disease, chronic diarrhoea, recurrent fracture, visual impairement, patent ductus arteriosus, developmental delay, horseshoe kidney, scalp eczema, right exotropia, hernia, renal tubular acidosis, vitamin D deficiency, hepatomegaly, conjugated jaundice.	Yes	No	Prednisolone, baricitinib, mycophenolate mofetil		No	
11	Trichohepatoenteric syndrome	17.5	Male	Low B cells, low IgG, A and M, poor vaccine responses.	Recurrent ear infections, recurrent respiratory infections, skin infection, conjunctivitis	Mouth ulcers, chronic diarrhoea, nocturnal enuresis, pancolitis, joint pain and hyperomobility, eczema, splenomegaly.	Yes	Yes	Mycophenolate mofetil		No	
12	Noonan syndrome	13.4	Male	Low IgG, absent MBL, common variable immune deficiency.	Recurrent respiratory infections, urinary tract infection.	Pulmonary stenosis, laryngomalacia, neurobehavioural problem, asthma, allergic rhinitis, type 1 diabetes mellitus, sleep problem, arthralgia, easy bruising, dyspraxia, sensory processing disorder, autism, learning difficulties, café au lait spots, multiple drug allergy, cyclical neutropaenia.	Yes	Yes			No	
13	Trisomy 22	15.8	Male	Low T cells, low IgG. Poor vaccine response.	Recurrent respiratory infections, recurrent urinary tract infections.	Left lung hypoplasia, imperforate anus, asplenia, total anomalous pulmonary venous drainage, pulmonary stenosis, left atrial isomerism, gut malrotation, nephrocalcinosis, ureteric dilatation, hypertension, ADHD, asthma.	Yes	Yes			No	
14	Trichothiodystrophy/ Cockayne syndrome complex	8.6	Female	Low IgG, intermittent neutropenia.	Recurrent respiratory infections, adenovirus gastroenteritis, *Enterococcus* urinary tract infection.	Trichothiodystrophy, icthyosis, myopia, esotropia, micropthalmia, horizontal nystagmus, cataract, delayed brain myelination, developmental delay, myoclonic movements, phototensitivity, osteosclerosis, raised alanine transaminase, kyphosis.	Yes	Yes			No	
15	Myhre syndrome	11.8	Male	Low IgG.	Recurrent respiratory infections, recurrent severe norovirus infection, purulent rhinitis, scalp fungal infection, conjuctivitis, dental abscess.	ADHD, global developmental delay, stiff joints, thumb deformity, sleep problem, coarctation of aorta, precocious puberty, vitamin D deficiency.	Yes	Yes			No	
16	Arboleda-Tham syndrome	13.0	Female	T cell lymphopaenia, low IgG, MBL deficiency, intermittent neutropenia, poor vaccine response.	Recurrent/persistent wet cough & respiratory infections, otitis media, mastoiditis, recurrent urinary tract infections.	ADHD, learning difficulty, diarrhoea, constipation, sleep problem, recurrent seizures, laryngomalacia, hypermobility, conductive hearing loss, myopia, vitamin D deficiency, humerus fracture, incontinence, mytochondrial cytopathy, swallowing problem, developmental delay, motor difficulties.	Yes	No			No	
17	Sideroblastic anaemia with B cell immunodeficiency, periodic fevers and developmental delay (SIFD syndrome)	4.9	Male	Low B cells, low IgG, A and M.	HHV6 viraemia, sepsis, skin and soft tissue infection.	Anaemia, splenomegaly, eczema, arthritis, chronic diarrhoea, hepatitis, developmental delay, refractive error, dactylitis, raised amyloid, thrombocytosis.	Yes	No	Anakinra, etanercept		No	
18	Supernumerary ring chromosome 20 syndrome	2.0	Male	T cell dysfunction, failure of T cell proliferation to mitogens, low CD8 and naive T cells, absent B cells, high NK cells, absent IgG, A and M, thombocytopenia.	Recurrent respiratory infections, sapovirus, rotavirus infection, MRSA colonisation, fungal urinary tract infection, *E. coli* sepsis.	Eczema, failure to thrive, congenital hypothyroidism, bilateral developmental dysplastic hips, bilateral small kidneys, pancreatic insufficiency, dermatitis, developmental delay, microphthalmia, bilateral cryptochordism, raised alanine transaminase, gastroesophageal reflux disease, recurrent diarrhoea, thrombocytopaenia.	Yes	Yes			Yes	NA
19	Kabuki syndrome	22.8	Female	Low IgG, undetectable IgA, thombocytopenia.	Bronchiectasis, recurrent respiratory infections, chronic glue ear, urinary tract infection, severe chicken pox, shingles.	Epilepsy, learning difficulty, horseshoe kidney, hyperextensibility, cystic liver with transaminitis, short stature, cleft palate, thrombocytopaenia	Yes	Yes			No	
20	Cartilage-Hair Hypoplasia	15.3	Male	T cell cytopenia, γδ T-cell expansion, very low TRECs, absent PHA response, anaemia.	Bronchiectasis, oral/oesophageal candidiasis, otitis media, shingles, norovirus gastroenteritis.	Fatty liver, hypocalcemia, chronic diarrhoea, biliary obstruction, malabsorption, iron deficiency anaemia, subcutaneous granulomata, arthralgia, sleep disorder, short stature, uveitis, intussusception.	Yes	Yes			Yes	Pneumonia
21	Sideroblastic anaemia with B cell immunodeficiency, periodic fevers and developmental delay (SIFD syndrome)	1.6	Female	Absent B cells, agammaglobulinaemia, severe anamia, recurrent red cell transfusion.	Norovirus gastroenteritis, *E. faecium* sepsis, rhinovirus pneumonitis.	Chronic diarrhoea, pulmonary hypertension, distral renal tubular acidosis, developmental delay, anaemia.	Yes	Yes	Anakinra	HSCT	Yes	Norovirus infection; global encephalitis with widespread infarction
22	Roifman syndrome	6.6	Female	Low B cells, low IgG, A and M.	Eczema herpeticum, shingles, norovirus gastroenteritis.	Cow's milk protein intolerance, eczema herpeticum, intrauterine growth retardation, dactylitis, developmental delay, right hemiplegia, hypermobility, acute disseminated encephalomyelitis.	Yes	No			No	
23	Chromosome 2p16.3 deletion	8.2	Male	Low IgG.	Recurrent respiratory and skin infections.	Tourette syndrome, Asperger syndrome, mouth ulcers, motor and sendory difficulties.	No	Yes			No	
24	Wiskott-Aldrich syndrome	21.8	Male	Low IgG, thrombocytopaenia.	Norovirus gastroenteritis, severe chicken pox, pinna cellulitis and pre-auricular abscess, chronic cough, pneumonia, *S. aureus* central line sepsis.	Recurrent scleritis, splenectomy, allergic rhinitis and eczema, recurrent arthralgia, thrombocytopaenia, glomerulonephritis.	Yes	Yes		Lentiviral gene therapy	No	
25	Wiskott-Aldrich syndrome	5.7	Male	Low IgG, thrombocytopaenia.	Recurrent respiratory infections, norovirus and sapovirus gastroenteritis, otitis media and externa, adenoviraemia, chicken pox, cradle cap, oral thrush, conjunctivitis.	Charcot-Marie-Tooth syndrome (asymptomatic), hepatitis, eczema, chronic diarrhoea, anal ulcer, speech delay, cow's milk protein allergy, thrombocytopaenia.	Yes	Yes		HSCT (complicated by grade 3 GVHD)	No	
26	Wiskott-Aldrich syndrome	3.1	Male	Low IgG, autoimmune thrombocytopenia.	EBV viraemia, *E. coli* and *Enterococcus* central line sepsis, *C. difficile* infection.	Eczema, anaemia.	Yes	Yes		HSCT	No	
27	Hyper IgE syndrome (STAT3 deficiency)	27.0	Female	Low IgA, poor vaccine responses, reduced Th17, raised IgE, reduced CD4+IL17+ and CD4+RO+IL17+.	Recurrent respiratory infections, skin and soft tissue infections and abscesses, otitis media and externa, orbital cellulitis, dental infection, mediastinal abscess, retropharyngeal abscess.	Paratracheal air cyst/diverticulum, pathological fracture, retention of deciduos teeth, pulmonary embolism and internal jugular thrombosis.	Yes	Yes			No	
28	Hyper IgE syndrome (STAT3 deficiency)	25.0	Female		MRSA recurrent skin and soft tissue infection and abscesses, fungal nail infection, recurrent otitis.	Eczema, scoliosis, joint hypermobility, retained primary teeth, fractures, asthma.	Yes	Yes			No	
29	Hyper IgE syndrome (STAT3 deficiency)	24.0	Male	IgE > 5000 kU/L.	Recurrent sinopulmonary infections, cradle cap, *S. aureus* skin and soft tissue infection and abscess.	Fracture, hypermobility, dermatitis/eczema, chronic rhinitis, osteopaenia, constipation, hypotonia, delayed motor milestones.	Yes	Yes			No	
30	Sideroblastic anaemia with B cell immunodeficiency, periodic fevers and developmental delay (SIFD syndrome)	1.3	Male	Low B cells and low IgG.	*S. epidermidis* central line infection.	Proximal renal tubulopathy, dilated pulmonary artery and aortic root with small ventricular septal defect, carnitine deficiency, chronic diarrhoea, pancreatic insufficiency, meconium ileus, developmental delay.	Yes	No	Etanercept		Yes	Covid-19; cerebral edema
31	Wiskott-Aldrich syndrome	2.8	Male	Normal immunology. Persistent and profound thrombocytopenia - probable autoimmune thrombocytopaenia (some increase in platelet count after steroid treatment, and IVIG; rapid loss of platelets following platelet transfusion)		Eczema, thrombocytopaenia.	No	No	Rituximab, prednisolone.		No	
32	Hyper IgE syndrome (STAT3 deficiency)	17.0	Female	IgE >2000 kU/L	Recurrent sinopulmonary infections, skin infections and abscesses, dental abscesses, lymphadenitis, lung abscess and empyema.	Pathological fractures, thoracic lumbar scoliosis, joint laxity, follicular hyperkeratoses/keratosis pilaris, eczema.	No	No			No	
33	MIRAGE syndrome	16.8	Male	Low B cells, low IgG, suboptimal vaccine responses.	Persistent wet cough.	Intrauterine growth restriction , glomerulonephritis, ambiguous genitalia, developmental delay, gastroesophageal reflux, gut dysmotility.	No	Yes			No	
34	Kabuki syndrome	9.2	Female	Low IgA.	Pyelonephritis, mastoiditis and recurrent otitis and respiratory infections.	Dysplastic kidneys, chronic kidney disease, hypertension, global developmental delay, bilateral dysplastic hips, joint hypermobility.	No	Yes			No	
35	Kabuki syndrome	6.6	Female	Slightly reduced CD8 and B cells, low IgG.	Pneumonia.	Complex congenital heart disease, developmental delay, developmental dysplasia of the hip, congenital hypothoyroidism.	No	No			No	
36	Roifman syndrome	8.0	Male	Low B cells, reduced T cell proliferation to PMA/ Ionomycin, low IgG, A and M, poor vaccine response (Diphtheria, Tetanus, Measles, Mumps).	Severe RSV pneumonia, enterovirus meningitis, recurrent respiratory infections, candidiasis.	Hypothyroidism, developmental delay, tracheobronchomalacia, laryngomalacia, spondyloepiphyseal dysplasia, hypermetromia, nystagmus, ventriculomegaly.	Yes	No			No	

ABBREVIATIONS

ADHD	Attention deficit hyperactivity disorder
EBV	Epstein-Barr virus
GVHD	graft versus host disease
HHV	human herpesvirus
HSCT	Haematopoeitic stem cell transplantation
Ig	Immunoglobulin
IVIG	intravenous immunoglobulin
MBL	mannose-binding lactate
NA	not available
PHA	phytohaemagglutinin
PMA	Phorbol myristate acetate
RSV	Respiratory syncytial virus
TREC	T cell receptor excision circles
